# The Strain Rate Sensitivity and Creep Behavior for the Tripler Plane of Potassium Dihydrogen Phosphate Crystal by Nanoindentation

**DOI:** 10.3390/mi12040369

**Published:** 2021-03-30

**Authors:** Jianhui Mao, Wenjun Liu, Dongfang Li, Chenkai Zhang, Yi Ma

**Affiliations:** 1 College of Mechanical and Electrical Engineering, Quzhou College of Technology, Quzhou 324000, China; 151548@qzct.net (J.M.); 151351@qzct.net (W.L.); 151566@qzct.net (D.L.); 152369@qzct.net (C.Z.); 2 College of Mechanical Engineering, Zhejiang University of Technology, Hangzhou 310014, China

**Keywords:** KDP, nanoindentation, hardness, strain rate sensitivity, creep, size effect

## Abstract

As an excellent multifunctional single crystal, potassium dihydrogen phosphate (KDP) is a well-known, difficult-to-process material for its soft-brittle and deliquescent nature. The surface mechanical properties are critical to the machining process; however, the characteristics of deformation behavior for KDP crystals have not been well studied. In this work, the strain rate effect on hardness was investigated on the mechanically polished tripler plane of a KDP crystal relying on nanoindentation technology. By increasing the strain rate from 0.001 to 0.1 s^−1^, hardness increased from 1.67 to 2.07 GPa. Hence, the strain rate sensitivity was determined as 0.053, and the activation volume of dislocation nucleation was 169 Å^3^. Based on the constant load-holding method, creep deformation was studied at various holding depths at room temperature. Under the spherical tip, creep deformation could be greatly enhanced with increasing holding depth, which was mainly due to the enlarged holding strain. Under the self-similar Berkovich indenter, creep strain could be reduced at a deeper location. Such an indentation size effect on creep deformation was firstly reported for KDP crystals. The strain rate sensitivity of the steady-state creep flow was estimated, and the creep mechanism was qualitatively discussed.

## 1. Introduction

As a typical multifunctional material, potassium dihydrogen phosphate (KDP) has attracted much attention for its outstanding photoelectric, piezoelectric and ferroelectric properties [1,2,3,4,5]. Large-size KDP crystals have been widely adopted in the frontier and important fields such as laser devices, information conversion, frequency conversion, nonlinear optics, etc. The ultraprecise machining process is the last critical procedure before the application of KDP crystals. In order to ensure the qualified service life and functional behavior of the device, good surface qualities, i.e., low surface roughness, flatness and negligible subsurface damage, are strictly required [6]. However, soft-brittle and deliquescent characteristics make KDP crystals among the most difficult-to-process materials. Consequently, fully revealing surface mechanical responses and their damage mechanism is necessary for improving ultraprecise machining technologies such as single-point diamond cutting, magnetorheological polishing and ultraprecision grinding.

In the last several decades, hardness values on different crystallographic planes of KDP crystals were widely investigated. Anbukumar et al. reported hardness values in the range of 1.57–1.77 GPa for the (001) plane under applied loads of 5–50 g by Vickers indentation [7]. Fang et al. studied the mechanical anisotropy of the (001) plane, observing that hardness was distributed between 1.3 GPa and 1.9 GPa using the Knoop indentation method [8]. Kucheyev et al. investigated the mechanical responses of (100) and (001) faces by spherical nanoindentation, and the average hardness values were 2.0 GPa and 1.6 GPa for the (100) and (001) faces, respectively [9]. Guo et al. studied the indentation size effect of nano-hardness on the (112) plane, which decreased from 2.61 to 1.44 GPa with increasing applied load from 0.5 to 8 mN [10]. Recently, the incipient plastic deformation in brittle KDP has attracted scientific interest at a very shallow depth by nanoindentation. Borc et al. studied elastic-to-plastic transition via pop-in events on the (001) and (100) faces of KDP crystals by Berkovich nanoindentation [11]. Zhang et al. studied incipient plasticity from the first pop-in event on the mechanical-polishing-damaged surface and nondamaged surface of KDP crystals [12].

Strain rate sensitivity (SRS) is a critical mechanical parameter that describes the strain rate effect on strength or hardness and can be adopted as an indicator of the ability of plastic and ductile deformation [13,14]. In recent years, SRS has been widely revealed by experimental methods in crystalline and amorphous alloys, by which the activation volume of the plastic unit, i.e., dislocation (for crystalline alloy) and the shear transformation zone (for amorphous alloy) could be experimentally estimated [15,16,17]. From the perspective of plastic mechanism, SRS detection is now a validated method to study the relationship between the inner structure and macroscopic deformation behavior, particularly ductility [18,19]. For brittle solids with covalent and ionic bonding, their fracture strength is stochastically distributed in a much wider range, which follows the well-known Weibull distribution [20]. Consequently, SRS and the plastic unit are rarely investigated in brittle solids such as ceramics because dislocation movement is negligible, and cracking is dominated during deformation [21].

Creep deformation is significantly important for materials suffering from long-term elastic stress at high temperatures. Creep damage is difficult to trace and seriously influences the service life and structural integrity of high-temperature components. At ambient temperatures, creep deformation could be negligible, particularly for high-melting-point materials, i.e., alloys, ceramics, concretes, etc. Beneficial to the extremely high testing accuracy, time-dependent deformation promoted by high stress could be captured by nanoindentation in a short duration (several to hundreds of seconds) at room temperature [22,23,24]. In light of the long-term pressure between abrasive particles and the surface, time-dependent plastic deformation should be taken into consideration during the mechanical polishing process. Zang et al. confirmed that creep deformation was pronounced on a KDP surface by a nanoindentation holding test at room temperature [25]. However, details of the creep behavior of KDP crystals such as the holding strain effect and size effect have rarely been unfolded.

In this work, we aim to study the characteristics of strain rate sensitivity and creep behavior on the tripler plane of a KDP crystal. In comparison to other crystalline orientations, the mechanical properties of the tripler plane were quite limited. Based on the variation of hardness with different strain rates, the strain rate sensitivity and activation volume of dislocation nucleation were determined. Relying on a spherical tip, the holding strain effect on creep behavior in a KDP crystal was systematically revealed. By adopting the self-similar Berkovich indenter, the indentation size effect on creep behavior was studied at a fixed holding strain. The creep mechanism of the KDP crystal was qualitatively discussed based on the strain rate sensitivity of the steady-state creep flow. 

## 2. Materials and Methods 

**Figure 1:**
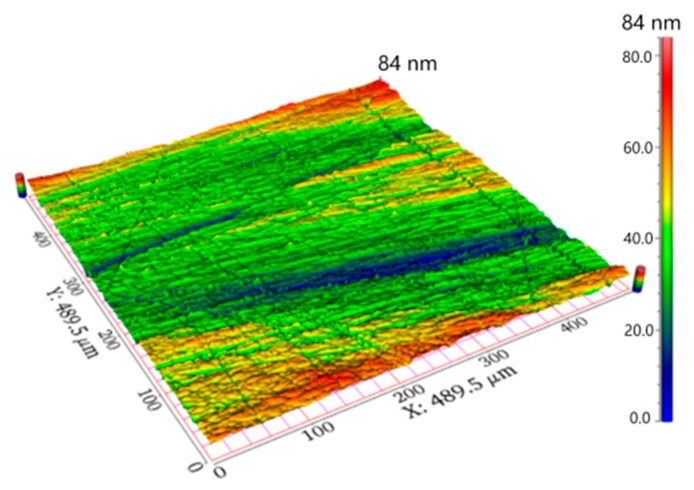
Three-dimensional surface morphology on the area of 480 × 480 μm^2^ by an optical profiler.

The KDP crystal was fabricated from aqueous solutions by the temperature reduction method and supplied by TianTong Company (Jiaxing, China), prepared with dimensions of 5 × 10 × 10 mm^3^ in height, length and width. The tripler face with a 10 × 10 mm^2^ area was polished by the traditional solution mixed with absolute ethyl alcohol and nanoscale silicon dioxide particles on a polyurethane (PU) pad on the Nanopoli-100 (NaiNuo, Hangzhou, China). After about 75 min of polishing, the tripler plane was carefully cleaned in anhydrous alcohol by ultrasonic cleaning. Using the optical profiler (Zygo Newview 5022, ZYGO, Bowen, PA, USA), the polishing performance was detected, as shown in Figure 1, whereby the surface roughness *R*_a_ was thus determined as 2.0 nm on the area of 490 × 490 μm^2^. Although scratches were not completely eliminated, the broad flat and smooth areas (length scale was larger than 150 μm) could ensure the testing reliability during the follow-up nanoindentation tests.

Nanoindentation testing was conducted on an Agilent Nano Indenter G200 at a constant temperature of 20 °C controlled by an air conditioner. At first, the elastic modulus and hardness were measured using the continuous stiffness module (CSM) method by a standard Berkovich indenter. Prior to the measurement, the tip area function was calibrated on fused silica. The maximum displacement was 2000 nm, and the strain rate was constant at 0.05 s^−^^1^. After the CSM measurement, the basic mechanical properties in the depth range from 0 to 2000 nm could be obtained. The strain rate sensitivity was estimated via the individual indentation method at a constant depth. Four different strain rates, i.e., 0.1, 0.03, 0.01 and 0.003 s^−1^, were adopted to study the strain rate effect on hardness, as well as the strain rate sensitivity value. In order to eliminate the tip bluntness effect at a shallow pressed depth and creep deformation during the long-time loading sequence, a medium pressed depth should be adopted for estimating strain rate sensitivity. Hence, the maximum pressed depth was set to 800 nm for the individual strain-rate-controlled tests. Furthermore, the rate-jump method by which the strain rate could be changed during the loading sequence was adopted to confirm the SRS result. For the rate-jump measurement, the initial displacement was set to 250 nm, and the displacement per strain rate was 200 nm. During the loading sequence, three different strain rates (0.1, 0.022 and 0.005) s^−^^1^ were successively conducted at the same indent.

Room-temperature creep deformations were detected by the constant load-holding method by a conical indenter with a spherical tip and a Berkovich indenter, respectively. For the spherical tip with an effective radius of 9.8 μm (by calibrating on fused silica and fitted by Herztian elastic theory), five peak loads of 5, 15, 50, 150 and 450 mN were adopted, and the loading rate was constant at 5 mN/s. The holding time was fixed at 500 s. For the Berkovich indenter, five peak loads of 1, 2, 5, 10 and 20 mN were utilized, and the loading rate was 1 mN/s. The holding time was 250 s. For all the tests, the unloading rate was equal to the loading sequence. The nanoindentation tests were launched until the thermal drift reduced below 0.05 nm/s. For ensuring the reliability of the experimental results, twelve individual indents were conducted for each case, and the interval between two adjacent indents was kept at least 50 times larger than the pressed depth. The residual impressions at the beginning of the creep test for both indenters under various peak loads were studied by a scanning electron microscope (SEM, sigma hv-01-43, St. Louis, MO, USA).

## 3. Results and Discussion

### 3.1. Strain Rate Sensitivity

**Figure 2:**
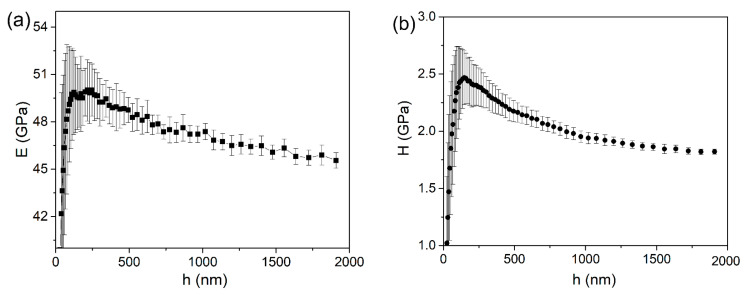
(**a**) Elastic modulus and (**b**) hardness plotted as a function of displacement by the continuous stiffness module (CSM) method.

Based on the CSM measurements, the elastic modulus and hardness at each pressed depth could be obtained, and their average values are shown in Figure 2. Due to the tip bluntness, the mechanical results by nanoindentation could be inaccurate at a shallow depth [26]. Clearly, both elastic modulus and hardness have good repeatability in different areas. At a deep penetration depth (larger than 500 nm), the error bars could be negligible. The common indentation size effect (ISE) on hardness also appeared for the tripler plane of the KDP crystal, although it was not significant. By increasing the displacement from about 200 to 2000 nm, the mean value hardness values decreased from 2.48 to 1.85 GPa. It should be noted that the mean elastic modulus value decreased from about 50 to 46 GPa as the indenter pressed into the maximum depth.

**Figure 3:**
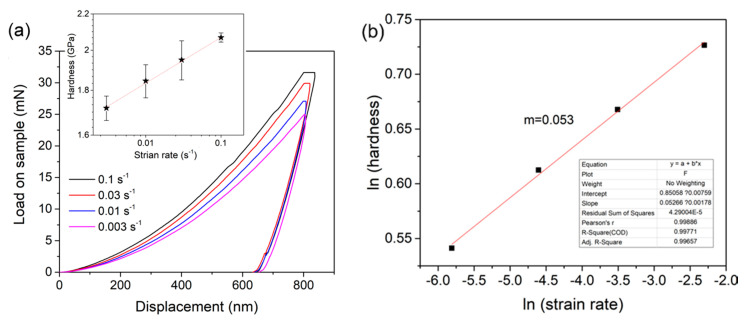
(**a**) Representative load versus displacement *P-h* curves by the four strain rates at the maximum pressed depth of 800 nm. The obtained hardness values under various strain rates are shown in the inset. (**b**) Log-log correlation between hardness and the strain rate. The strain rate sensitivity *m* could thus be estimated by linear fitting.

Figure 3a shows the representative *P-h* (applied load versus displacement) curves with various strain rates at the maximum depth of 800 nm. Obviously, higher loads were required to attain the same displacement under a faster loading sequence, which indicates the stronger resistance to plastic deformation, namely the higher hardness. By increasing the strain rate from 0.001 to 0.1 s^−1^, the average hardness increased from 1.67 to 2.07 GPa, as shown in the inset. The strain rate sensitivity (SRS) exponent *m* can be determined via [14]:

(1) $$m=\frac{\partial \ln H}{\partial \ln\overset{\cdot }{\varepsilon }}$$

Figure 3b shows the log-log correlation between hardness and the strain rate for the tripler plane, in which hardness almost linearly increased with increasing strain rate. By linearly fitting, the SRS was computed as 0.053.

**Figure 4:**
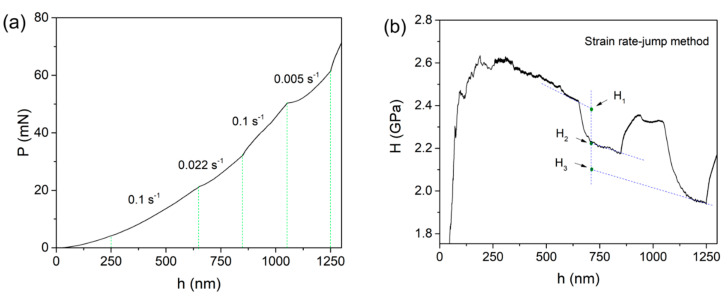
(**a**) Loading sequence of the rate-jump method. (**b**) Variation of hardness under different strain rates.

**Figure 5:**
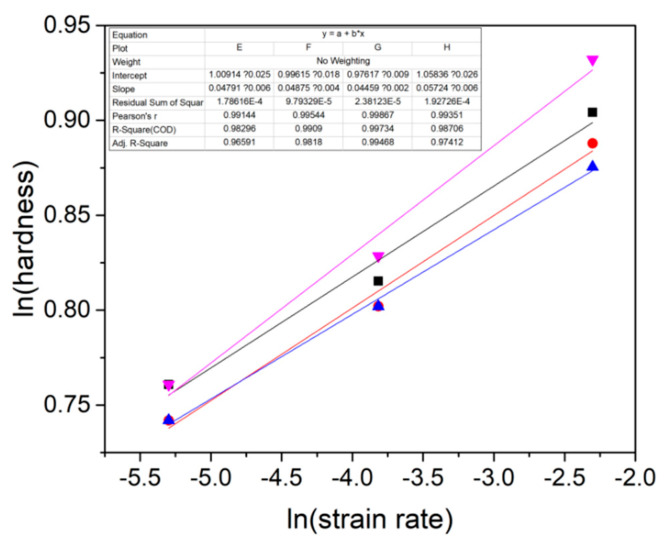
Log-log correlation between hardness and strain rate detected at four independent places by the rate-jump method. Strain rate sensitivities could be estimated by linear fitting.

Figure 4a shows the representative loading segment of the rate-jump experiment on the tripler plane. Figure 4b shows the corresponding hardness under each strain rate, which was plotted with the displacement. Due to the evident indentation size effect, average hardness was unable to be adopted. By using Maier’s method, the hardness values under various strain rates could be deduced at the same displacement [27]. Figure 5 shows the log-log correlation between hardness and the strain rate by the rate-jump method for four independent indents. By linear fitting, the strain rate sensitivities were estimated as 0.049, 0.048, 0.045 and 0.057. Hence, the average SRS was about 0.05, which validated the reliability of the SRS detection in this work.

In general, a higher *m* indicates greater resistance to localized deformation. For example, *m* is always an order of magnitude lower in brittle metallic glass (at the magnitude of 10^−^^4^~10^−^^3^ or even minus) than ductile metals after rate-jump nanoindentation [28,29]. In this work, the soft-brittle KDP has a high SRS value, which could be comparable to ductile copper and aluminum and much higher than well-known new-structure alloys such as metallic glass and high-entropy alloys [28]. To the best of the author’s knowledge, this is the first report on detecting SRS for KDP crystals. Only in a few reports have researchers paid attention to the loading rate effect on elastic-to-plastic transition, i.e., the first pop-in event for KDP crystals [11]. The strain rate-concerned hardness has never been studied in KDP hitherto. In the current work, we do not intend to study the plastic removal mechanism of KDP crystals at the nanoscale. The results herein indicate that resistance to plastic or brittle deformation of the KDP crystal is strongly related to the strain rate. The high SRS value suggests that the machining performance such as surface roughness and material removal rate of the KDP crystal could be influenced by rotation speed during the grinding or polishing processes. Furthermore, the high strain rate sensitivity suggests that plastic deformation could occur at the microscale for the brittle KDP single crystal, which was validated in the small-size sample for ionic- and covalently bonded hard material [30]. Hence, it could be necessary to estimate the activation volume for plastic deformation [31].

According to Wei et al.’s work [13], the activation volume v^*^ for plastic deformation could be expressed as:

(2) $$v^{*}=T\frac{k_{B}}{m\tau_{y}}$$

Here, *τ_y_* is the critical shear stress in the traditional tensile or compressive tests, which has an empirical correlation of *τ_y_* ≈ *H*/3√3. *k*_B_ is the Boltzmann constant, and *T* is the testing temperature. The average hardness value (2.0 GPa) in the CSM of which the strain rate was 0.05 s^−^^1^ at a depth of 800 nm was adopted. Once *H* and *m* were determined, the activation volume for plastic deformation could be concomitantly calculated as 169 Å^3^. The activation volume for plastic deformation in the KDP crystal was evidently larger than the previous reports in metals and alloys, of which the size was at the atomic scale [32,33,34]. The sources of dislocation nucleation could be assumed to be point-like defects such as impurities and vacancies as atomic-scale v^*^ were detected [35]. In comparison, the v^*^ for the tripler plane was in the length scale of 0.55 nm, which was estimated from the cube root of v^*^, approaching the size of the KDP unit cell. The cooperative migration of several atoms, i.e., the atomic cluster, could be the mechanism of dislocation activation. Accordingly, a larger v^*^ indicates higher activation energy for plastic deformation. From the perspective of the deformation mechanism, the slip systems in the brittle KDP crystals were quite limited. In KDP crystals, cracking deformation is initiated at the “fertile place” with surface damage, structure defects and pre-existing dislocations and then rapidly develops to a catastrophic cracking fracture. The result herein of the large-size v^*^ explains the deformation feature of KDP crystals that dislocation activation and movement are difficult to occur.

### 3.2. Room-Temperature Creep Behavior

**Figure 6:**
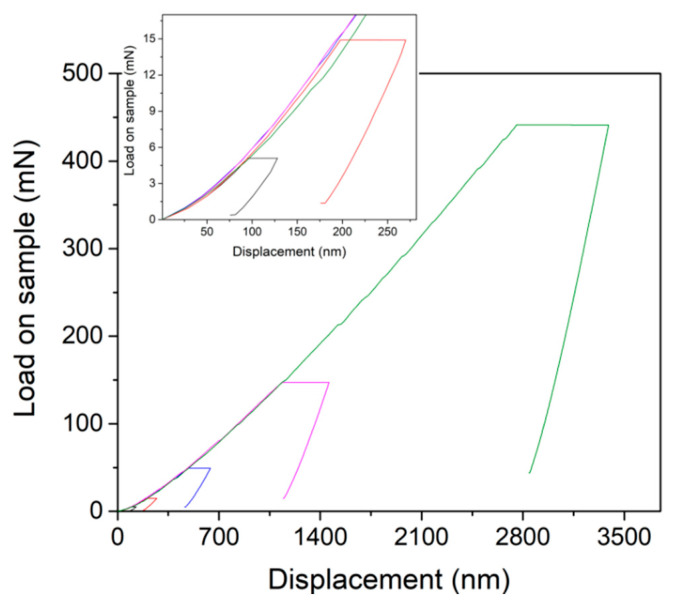
Representative creep *P-h* curves at different holding loads from 5 to 450 mN under a spherical tip of a 9.8 μm radius.

Figure 6 shows the representative creep *P-h* curves at various peak loads under the spherical tip. Obviously, time-dependent plastic deformation occurred during holding stages even for small peak loads, as shown in the inset. Although atomic diffusion of the KDP crystal was unable to occur at room temperature, the high stress beneath the indenter could also facilitate the creep flow, particularly for the materials with a low melting point or soft nature [36,37]. Notwithstanding, it was held in the plastic region (the minimum holding strain was beyond the elastic limit of KDP crystal), the time-dependent plastic deformation herein could also be defined as creep flow.

**Figure 7:**
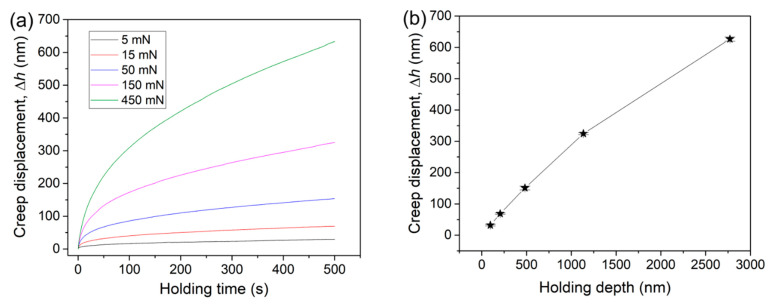
(**a**) Creep displacements during the holding stages for different holding loads (both the onsets of the holding time and creep displacement were set to zero for clear observation). (**b**) Total creep displacements at the ending of duration at various holding depths.

Figure 7a shows the corresponding creep displacements during the holding stage, in which both the holding time (*X*-axis) and creep displacement (*Y*-axis) were set as zero for a clear view. For the KDP crystal, the typical nanoindentation creep behavior could be discerned so that only two creep stages, i.e., transient and steady-state creep flows, appeared. The third stage, namely the failure stage, was missed because the core region, which attained the yielding condition, could continuously expand under the indenter. In comparison to conventional creep deformation, the transient stage during nanoindentation holding was much shorter. For the KDP crystal, the transient stage could be distinguished in the initial 50 s, which seemed to be independent of the peak load. At this creep stage, displacement dramatically increased with holding time, while its increasing rate precipitously dropped. Approximately, creep displacement increased stably with the holding time as the duration exceeded 50 s. During the steady-state stage, creep displacement could almost linearly increase with the holding time. It was reported that a discontinuous displacement increase could appear in some kinds of brittle alloys and ceramics during nanoindentation holding [35,38], which suggests the delayed pop-in events during the holding stage at room temperature. The creep flow curves herein were smoother and more continuous, suggesting that the creep deformation of the KDP crystal should be homogeneous. 

**Figure 8:**
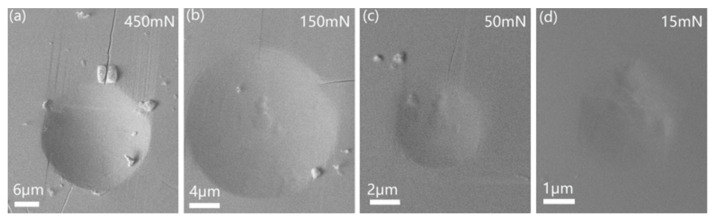
SEM images of residual impressions at peak loads of (**a**) 450, (**b**) 150, (**c**) 50 and (**d**) 15 mN under a 9.8 μm spherical tip.

By increasing the applied peak load, creep deformation was more pronounced for both creep stages. The total creep displacements at the end of the holding stage were carefully recorded for each test. As shown in Figure 7b, the total creep displacements nearly linearly increased with increasing holding depth. The enlargement of creep displacement could be due to several extrinsic and inner reasons. Extrinsically, both the holding strain and deformation region beneath the indenter were simultaneously increased with increasing holding load or depth for the spherical tip. It is well recognized that a higher holding strain enhances creep deformation by the traditional uniaxial method. On the other hand, creep displacement could be proportional to the deformation region (mainly the plastic region) beneath the indenter [39], provided that the creep resistance of the KDP crystal is size independent. Intrinsically, plastic or brittle deformation was more severe at higher loads under the spherical tip. As a result, the more agitated atomic structure could provide more fertile places for the occurrence of creep deformation. Figure 8 shows the morphologies of residual impressions at the beginning of the creep test at various peak loads (the noted residual impression at 5 mN was too weak to be discerned). For the high-stress loading at 450 mN, mainly cracks and a lot of twinning patterns appeared, as shown in Figure 8a. By decreasing the applied loads to 150 and 50 mN, twin deformation was not observed, and a very tiny crack was detected, as shown in Figure 8b,c. As the peak load reduced to 15 mN, crack and twin deformation were both missed. We are convinced that both the amount and size of the cracks were enhanced at the onset of the holding stage under higher peak loads. It could be expected that more severe stress concentration and active atoms appear around the crack tip, where creep flow can easily occur.

**Figure 9:**
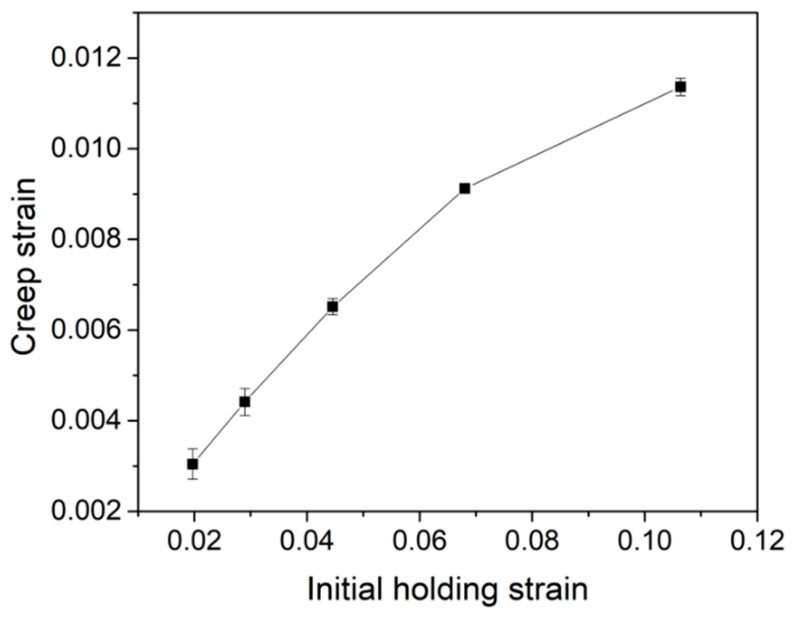
Total creep strains were plotted as a function of the initial holding depth.

To better study the creep behavior of the KDP crystal, the creep displacement could be replaced by creep strain under various holding strains. Creep strains under spherical indenter were calculated by [40]:

(3) $$0.2\left(\alpha -\alpha_{0}\right)/R$$

where *a* and *a*_0_ are the contact radii at the beginning and end of the holding stage, respectively. α is equal to $\sqrt{2Rh_{c}}$, where *h*_c_ is the contact depth defined as *h*_c_ = *h* − *εP*/*S*, ε = 0.75 and *S* is the stiffness deduced from the unloading curve. *R* is the effective spherical tip radius. Figure 9 shows the calculated total creep strains, which were plotted with initial holding strains. Clearly, creep deformation occurred more severely at a higher initial holding strain. Even under a ~2% holding strain, the 500 s creep deformation was pronounced, which attained about 3%. Creep strain was beyond 10% under the 10% initial holding strain. It should be mentioned that the creep deformation is actually influenced by both applied load and strain. The result in Figure 9 still contains both the effects of applied load and strain under nanoindentation. By changing the indenter type, creep deformation could be varied, even though the applied load or applied strain is the same. This observation indicated that the KDP crystal has a weak creep resistance even at room temperature. The creep deformation should be seriously considered during long-term polishing processes, during which the abrasive particles bring a similar effect to the nanoindentation holding test. Beneficial to the homogeneous creep deformation, the surface quality of the KDP crystal could be elevated by increasing the contact time between abrasive particles and the KDP surface. The brittle material removal manner of KDP could be alleviated by introducing more creep deformations.

**Figure 10:**
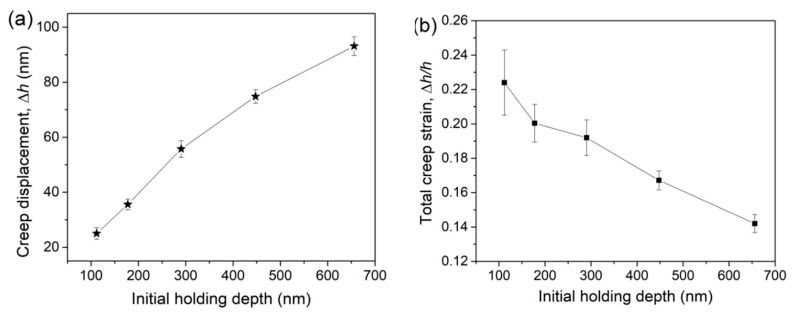
(**a**) Total creep displacement after 150 s holding stages at various holding depths by the Berkovich indenter. (**b**) Total creep strains were plotted as a function of initial holding depth.

Under spherical nanoindentation, both the holding strain and degree of plastic deformation were increased with increasing holding depth. By adopting the self-similar Berkovich indenter, under which the characteristic plastic strain is 7.1% [40], the indentation size effect on creep deformation could be studied. Figure 10a shows the total creep displacement ∆*h* after a 250 s duration under various holding depths. Similar to the spherical indentation holding test, creep displacement nearly linearly increased with initial holding depth. By simply defining the creep strain under Berkovich indenter as ∆*h/h* (*h* is the initial holding depth), the correlation between creep deformation and holding depth could be obtained as shown in Figure 10b. Under the same holding strain of 7.1%, creep strain gradually decreased from about 22.5% to 14% as holding depth increased from about 105 to 650 nm. Obviously, creep resistance was enhanced at a deeper location.

**Figure 11:**
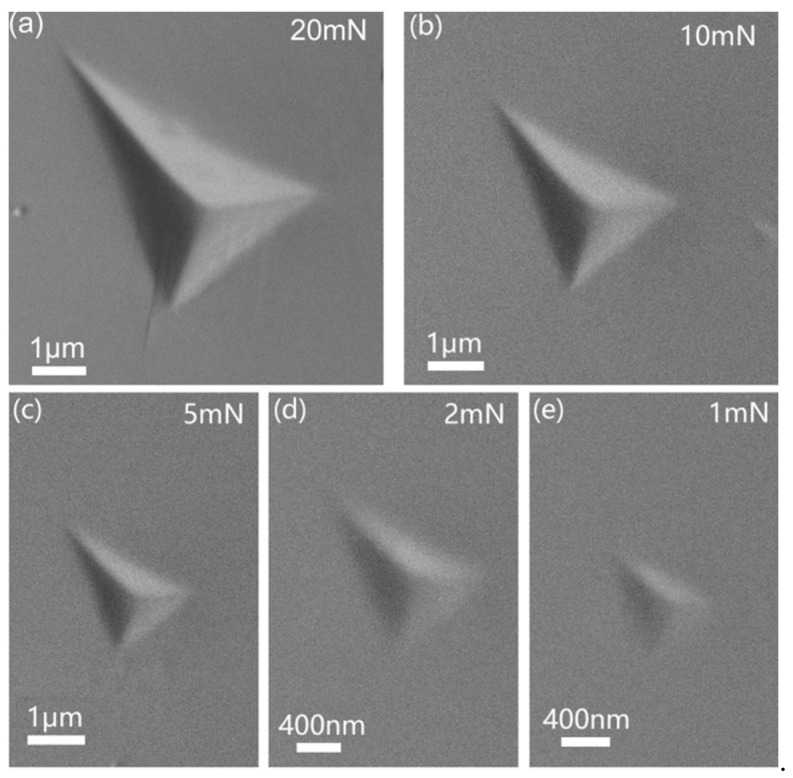
SEM images of residual impressions at various peak loads (**a**) 20, (**b**) 10, (**c**) 5, (**d**) 2 and (**e**) 1 mN at the beginning of creep holding under the Berkovich indenter.

Figure 11 shows the representative surface morphologies of residual impressions at the beginning of the creep holding segment for the five different peak loads. Only twin deformation and the crack were not observed.

From the perspective of structure damage, the mechanical polished KDP surface could be regarded as structure heterogeneous due to the appearance of surface and subsurface damage layers. In addition, the deliquescent nature of the KDP crystal might result in the surface alteration for long-term exposure to air. Hence, surface damages could be the critical reason for the detected indentation size effect on creep resistance. It could be expected that the creep strain would tend to be stable by further increasing holding depth under the self-similar Berkovich indenter.

**Figure 12:**
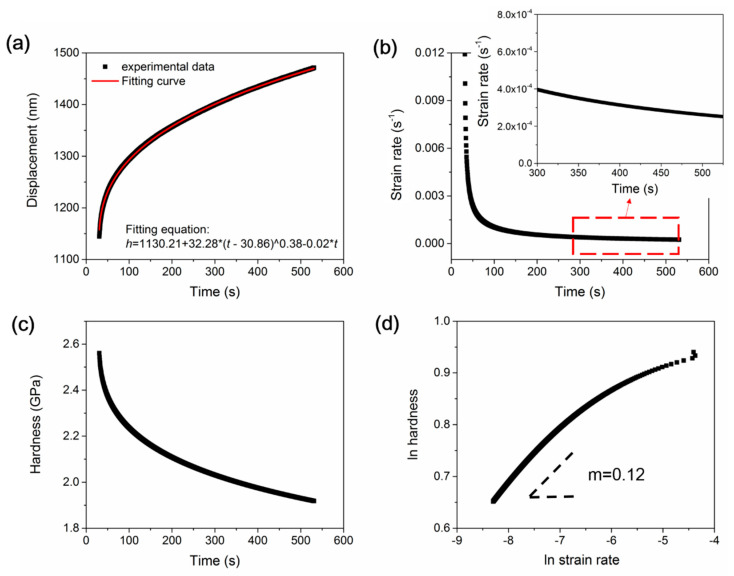
(**a**) The typical creep flow under 150 mN by 9.8 μm spherical tip was well fitted by an empirical law. (**b**) The changes of strain rates during the holding stage. (**c**) The changes of hardness during the holding stage. (**d**) The correlation between hardness and strain rate during the holding stage, and strain rate sensitivity could be estimated from the steady-state creep segment.

According to conventional creep deformation, the stress exponent *n* or strain rate sensitivity *m* (*m =* 1/*n*) could be indicative of the creep mechanism at high temperatures [41]. At high temperatures, dislocation movement could be dominated during creep flow for alloys as *m* falls in the range between 0.1 and 0.3. A higher *m* reflects stronger atomic activity at high temperatures. Here, we adopted the creep deformation under 150 mN holding under the spherical tip for calculating the strain rate sensitivity, of which the holding depth could be comparable to the rate-jump measurement in Figure 3a. As shown in Figure 12a, the representative creep curve was well fitted by an empirical law [24]:

*h*(*t*) = *h*_0_ + *a*(*t* − *t*_0_)^b^ + *kt* (4)

where *h*_0_ and *t*_0_ are the displacement and time at the beginning of the holding stage, and *a*, *b* and *k* are the fitting constants. Based on the creep fitting line, the variation of creep strain rate during the holding stage could be computed by:

(5) $$\dot{\varepsilon }\;=\;\frac{1}{\sqrt{A}}\frac{d\sqrt{A}}{dt}$$

where *A* is the contact area equal to $2\pi Rh_{c}$ in the plastic region. As shown in Figure 12b, the creep strain rate dramatically reduced from about 0.01 to 0.0015 s^−1^ at the transient stage. It then gradually decreased to about 2.5 × 10^−4^ s^−1^ at the end of the holding stage. In the plastic region, the mean pressure is also defined as hardness for the spherical tip, which is

(6) $$H=\frac{P}{2\pi Rh_{c}}$$

Figure 12c shows that hardness or mean pressure decreased from about 2.55 to 1.9 GPa during the whole 500 s holding stage. Once the strain rate and hardness during the holding stage were calculated, strain rate sensitivity could be determined according to Equation (1). Figure 12d shows the log-log correlation between hardness and the strain rate. By linear fitting on the steady-state creep segment, *m* could be obtained as 0.12. Following this method, we addressed six randomly selected creep curves under 150 mN holding, and the obtained SRS ranged from 0.1 to 0.13.

It should be emphasized that the SRS value of the nanoindentation creep flow could be greatly tied to the testing conditions such as holding time, loading rate and tip configuration [42]. It has been widely observed that *m* is increased with increasing holding time [42]. As shown in Figure 8d, the slope between hardness and the strain rate was evidently lower at the transient stage in comparison to the steady-state segment. Generally, the distinction of *m* at the two creeps stage could be more than an order of magnitude. In Zhang et al.’s work, *m* increased from about 0.035 to 0.07, and holding time merely increased from 5 to 60 s on the (001) plane of the KDP crystal [25]. Regardless of the crystalline orientation difference, the significantly longer duration could be the main reason for the higher SRS value on the tripler plane herein. It is worth mentioning that the *m* of the steady-state creep flow was two times that of the rate-jump method on the tripler plane in Figure 3b. Besides the aforementioned holding time effect, deformation manner could also be an important reason for the variation of *m*. Generally, the effective deformation strain rate $\dot{\varepsilon }$_i_ under nanoindentation could be much higher than that of the uniaxial compressive method, and there is an approximate transition relationship as [43]:

(7) $${\dot{\varepsilon }}_{u}\sim 0.09{\dot{\varepsilon }}_{i}$$

By this analysis, equivalent strain rates by the rate-jump method were 2.7 × 10^−4^ to 9 × 10^−3^ s^−1^, by which quasi-static deformation occurred. For steady-state creep deformation under the spherical tip, the equivalent strain rate was ~2 × 10^−5^ s^−1^. Such a low strain rate conformed well to the deformation feature of creep flow or stress relaxation. Clearly, brittle or localized manners would be dominated in the quasi-static deformation and homogeneous flow occurring in creep deformation for KDP crystals. Hence, it is reasonable that *m* by the rate-jump method could be much lower than the creep method as a higher *m* reflects the ability of the resistance to localized deformation. 

In a previous report [25], the nanoindentation duration on KDP crystals was short, and creep deformation did not fully turn into the steady-state stage where the creep mechanism is always discussed. In our previous work, we observed that the length and amount of the pre-existing cracks were unchangeable during the steady-state creep deformation for another soft-brittle material at room temperature [44]. It provided direct evidence that crack generation and propagation were unable to occur under such a low creep strain rate. Furthermore, we assumed that atom diffusion between the indenter and contact surface was also impossible, for the mobility of atoms and clusters at room temperature is insufficient. Hence, we could be convinced that dislocation movement, including the agitation of pre-existing dislocation and newly activated ones, would be dominating in the creep deformation for the tripler plane of a KDP crystal. The obtained *m* was between 0.1 and 0.3, which further suggested dislocation movement, i.e., dislocation climb and glide induced time-dependent plastic deformation for the KDP crystals under nanoindentation.

## 4. Conclusions

In summary, instant and time-dependent plastic deformations on the tripler plane of a KDP crystal were systematically studied relying on nanoindentation technology. Elastic modulus and hardness were slightly decreased with increasing pressed depth at the nanoscale. By increasing the strain rate, hardness evidently enhanced at the same depth, and accordingly, the strain rate sensitivity was obtained as 0.053. Under the spherical tip, creep deformation severely occurred within a 500 s duration. The greatly enhanced creep deformation was mainly due to the enlarged holding strain at a deeper location. By adopting the self-similar Berkovich indenter, total creep strain could be gradually reduced with increasing holding depth. It was indicated that creep resistance could be weakened at the surface or subsurface damage layer for the KDP crystal. The strain rate sensitivity of the steady-state creep was also estimated as 0.12. Dislocation movement might be the dominating creep mechanism for the KDP crystal under high-stress holding at room temperature. The current work firstly investigated the strain rate sensitivity and indentation size effect of creep deformation in a KDP crystal.
